# Assessing MRI interpretability of the orbit, paranasal sinuses, and nasopharynx in cochlear implant patients

**DOI:** 10.3389/fneur.2025.1636128

**Published:** 2025-07-31

**Authors:** M. C. Ketterer, P. Arnold, A. Aschendorff, S. Granitzer, M. Reich, A. K. Rauch, T. Hildenbrand, S. Arndt, L. Fries

**Affiliations:** ^1^Department of Otorhinolaryngology, Medical Center University of Freiburg, Faculty of Medicine, University of Freiburg, Freiburg, Germany; ^2^Department of Radiology, Medical Center University of Freiburg, Faculty of Medicine, University of Freiburg, Freiburg, Germany; ^3^Oticon Medical, Vallauris, France; ^4^Eye Center, Faculty of Medicine, Albert-Ludwigs University Freiburg, Freiburg, Germany

**Keywords:** magnetic resonance imaging, artifact, cochlear implant, orbital, paranasal sinus

## Abstract

**Objectives:**

Due to a growing focus on cost-effectiveness in healthcare, safety concerns with CI and the known limitations in image quality, there is an increasing need for well-considered indications before performing magnetic resonance imaging (MRI) in CI (cochlear implant) patients. This study aims to evaluate, for the first time, the clinical utility and limitations of MRI in CI patients for orbital, paranasal, and nasopharyngeal assessments.

**Materials and methods:**

CIs were positioned and fixed with bandaging around the head of a test subject at varying angular positions (90°, 120°, and 135°), both unilaterally and bilaterally, with and without the magnet in place. MRI acquisitions included T1-MP-RAGE, T2-TSE, T1-TIRM, and DWI sequences of a 3 Tesla MRI scanner. The MRI images were reconstructed three-dimensionally, and the resulting artifacts were analyzed to determine the interpretability of the predefined orbital, paranasal, and nasopharyngeal structures.

**Results:**

Image quality was categorized into four levels of restriction. It was observed that orbital MRI diagnostics in the required sequences (T1, T2, and DWI) are feasible even in patients with bilateral CIs with magnets *in situ*. Regarding the paranasal sinuses, artifacts affected the sphenoid sinus and parts of the ethmoidal cells; however, as expected, the interpretability improved significantly without the magnet. The nasopharyngeal space, particularly in patients with bilateral CIs and magnets *in situ*, could be evaluated only with difficulty or was largely not assessable.

**Conclusion:**

This study offers insights into the predictive factors influencing the interpretability of MRI scans for the orbit, paranasal sinuses, and nasopharynx in CI patients. In particular, for the sphenoid sinus and nasopharynx, it is strongly advised to consult the responsible CI center before undergoing an MRI examination. This consultation helps assess the necessity of the MRI and, if required, consider the removal of the implant magnet.

## Introduction

In recent years, significant progress has been made in enabling cochlear implant (CI) patients to undergo magnetic resonance imaging (MRI). Advances in CI technology have led to substantial improvements, allowing MRI scans of up to 3 Tesla. However, the quality of cranial MRI in CI patients remains considerably affected by implant-related artifacts (1–3). This limitation persists for intracranial MRI assessments, as demonstrated by our recent research, even when the CI magnet is removed (3).

MRI of the orbit, paranasal sinuses, and nasopharynx is important for specific indications, particularly in adolescence and adult patients. For example, MRI is crucial for diagnosing orbital masses such as lymphoma, carcinoma, or pseudotumor before biopsy or excision (4–7). While paranasal MRI is less frequently performed, it is invaluable for diagnosing malignancies like adenocarcinoma, squamous cell carcinoma and lymphoma (8), as well as for benign tumors like inverted papillomas (9–11). Furthermore, male adolescents with suspected enlarged adenoids should undergo angio-MRI of the nasopharynx to assess nasopharyngeal masses, such as juvenile nasopharyngeal angiofibromas. This imaging is crucial for planning pre-biopsy embolization and preventing potentially life-threatening hemorrhagic complications (12, 13).

With the increasing focus on cost-effectiveness in healthcare and previous studies underscoring the importance of well-founded clinical indications for MRI (14), this study is the first to investigate whether MRI of the orbit, paranasal sinuses and nasopharynx is of additional benefit in patients with CI.

## Methods

This study is based on and is complementing our research group’s recent study (3), but with the key difference that MRI was acquired from one healthy male adult volunteer who provided informed consent instead of cadaver heads. The volunteer did not report any discomfort during the MRI scans, which were performed in a single session.

The study was approved by the Ethics Committee of the University Hospital of the Albert-Ludwigs University of Freiburg (Approval Number: 24-1178-S1) and registered in the German and Freiburg Clinical Trials Register (DRKS Number: 00034859; FRKS Number: 005200).

The CI, provided by Oticon Medical, was positioned and fixed around the head of the participant with bandaging. Implants were placed at 90°, 120°, and 135° angles, as described in Arnold et al. (3) for intracranial MRI assessment. Evaluations were conducted for both unilateral and bilateral conditions, with and without the CI magnet. Our previous publication is demonstrating an illustration of the evaluated implant angles (3). The present research constitutes a pilot study.

In contrast to Arnold et al. (3), this study focused on visualizing the orbit, paranasal sinuses, and nasopharynx as extracranial anatomical structures, using a categorical visibility scale dividing into four categories (0–25%, 25–50%, 50–75%, and 75–100%) as established in the previous study.

All MRI scans were performed with a 3 Tesla MRI scanner (MAGNETOM Prisma, Siemens Healthcare, see Table 1 for technical data) with a maximal amplitude: of 139 mT/m8 and a maximal slew rate of 346 T/m/s8. The scans were independently analyzed by a radiologist with neuroradiology expertise, in collaboration with two head and neck/CI surgeons. Additionally, an ophthalmologist with over 5 years of neuro-ophthalmology experience focused on the orbit’s interpretability, while a head and neck surgeon with over 15 years of experience in rhinology and skull base surgery analyzed the paranasal sinuses, skull base, and nasopharynx. All experts evaluated the images independently, and the decision regarding representability, as shown in the table, was made unanimously by consensus.

**Table 1:** MRI sequence parameters, including voxel size, TR (time of repetition), TE (time of echo), and acquisition time, were selected as described by Arnold et al. (3).

Sequences	Voxel size	TR	TE	Acquisition time
3D T_1_ MP-RAGE	1.0 × 1.0 × 1.0 mm^3^	2,300 ms	2.26 ms	3:54 min
2D T_2_ W TSE	0.4 × 04 × 5.0 mm^3^	6,440 ms	110 ms	2:49 min
T_1_W-TIRM	0.7 × 0.7 × 5.0 mm^3^	2,000 ms	9 ms	2:54 min
DWI	0.6 × 0.6 × 5.0 mm^3^	3,500 ms	85 ms	0:47 min

MP-RAGE, magnetization prepared rapid gradient-echo; TSE, turbo spin echo; DWI, diffusion weighted imaging; TIRM, turbo inversion recovery magnitude.

These regions were evaluated and reconstructed in 3D, assessing visibility despite artifacts caused by the implant and magnet.

## Results

### Orbit

**Figure 1:**
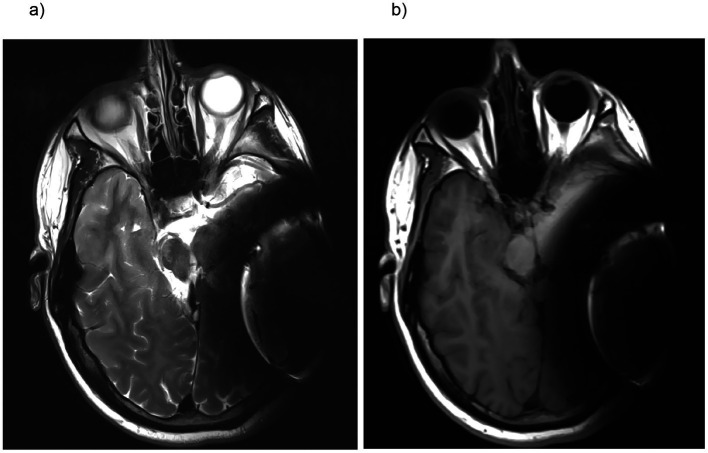
MRI (magnetic resonance imaging) in T2-weighted sequence **(a)** and T1-weighted turbo inversion recovery magnitude (TIRM) images **(b)** in a unilateral CI with a magnet positioned at a 120° angle. As shown, both the contralateral and ipsilateral orbits are fully visible and assessable.

**Figure 2:**
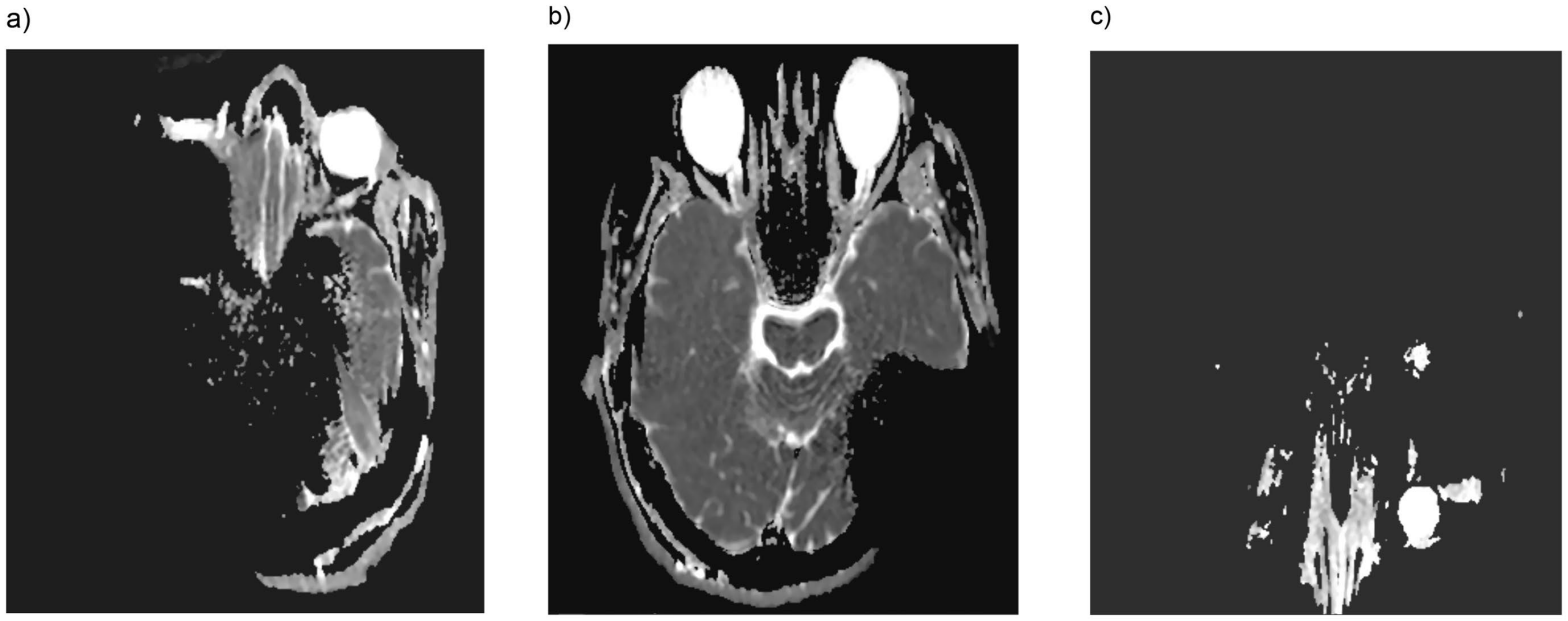
In DWI (diffusion weighted imaging) orbital accessibility for 135° cochlear implant positioning was impeded with the magnet in place unilaterally **(a)** or bilaterally **(c)**, whereas without the magnet **(b)**, the orbit was clearly visible and accessible.

The orbit was visible and assessable under the examined conditions for T1, T2 and TIRM sequences (see Figure 1), even in bilaterally implanted patients with the magnet *in situ* (Table 2). The eyeball, as well as the straight and oblique extraocular muscles, the optic nerve, the medial bony boundary to the ethmoid bone (lamina papyracea), the lateral boundary, the orbital floor, were clearly visible and assessable in the T1 and TIRM sequences. However, in some sequences, particularly T1, the orbital roof was not fully assessable, leading to a downgrade in visibility to category 2. Nevertheless, in DWI (diffusion weighted imaging) the orbit was not clearly assessable ipsi- and contralaterally especially in 90° and 120° positioned CI and in bilateral CI condition with magnet *in situ*, while in 135° positioned CI without magnet the orbit was accessible (see Figure 2).

**Table 2:** Interpretability of the sequences T1, T2, TIRM, and DWI for the following anatomical structures: ipsilateral and contralateral orbit, maxillary and frontal sinus (ipsilateral and contralateral), ethmoidal cells, sphenoid sinus, clivus, and nasopharynx.

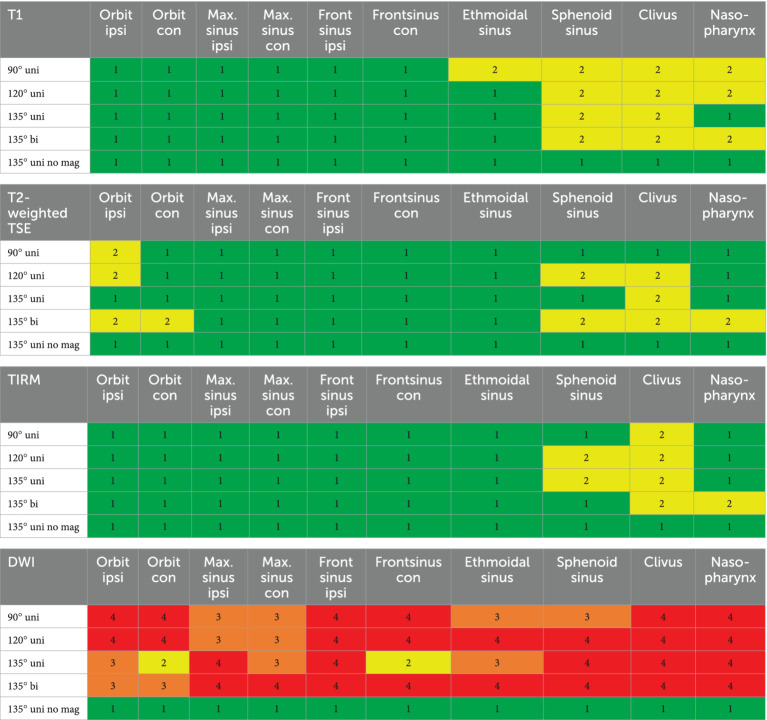

We established four different categories. Category 1 (green): clear evaluation is possible. Category 2 (yellow): mild to moderate limitations are present. Category 3 (orange): considerable limitations exist. Category 4 (red): no interpretability, as the image is entirely obscured by artifacts.

### Paranasal sinuses

**Figure 3:**
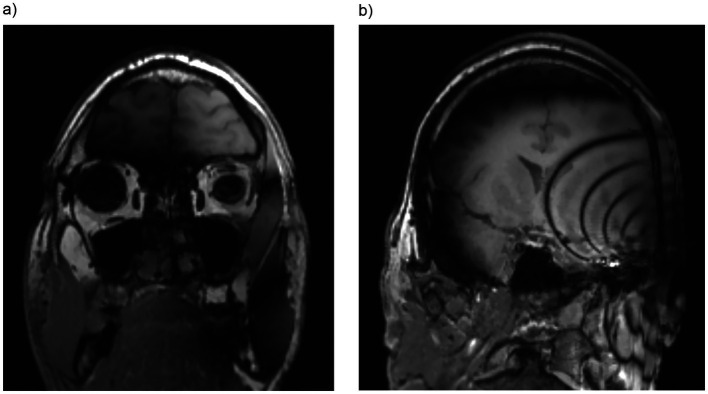
Both maxillary sinuses are fully visible and assessable in T1-weighted sequence **(a)** in a unilateral CI with a magnet positioned at a 90° angle. Furthermore, T1-weighted sequence **(b)** in a unilateral CI with a magnet positioned at a 120° angle demonstrating that the sphenoid sinus is only partially assessable. Coronal images are reconstructed from the volumetric T1 MP-RAGE sequence.

The evaluation of the paranasal sinuses was conducted as follows: the maxillary, frontal, and ethmoid sinuses were assessed separately for each side. The ethmoidal cells were evaluated as a single unit, while the sphenoid sinus was considered a single unit, as an intersphenoid septum is not always present anatomically. As demonstrated in Table 2 and Figure 3, the included sequences allowed for a clear diagnostic evaluation of the maxillary and frontal sinuses, as well as the ethmoidal cells, except for DWI. The nasal cavity was also clearly visible in T1 and T2 sequences and unaffected by artifacts. However, the sphenoid sinus was partially obscured by artifacts, particularly in bilateral CI condition. After magnet explantation, the sphenoid sinus became assessable even in bilateral CI cases.

The clivus, an anatomical structure in the central skull base, was evaluated separately (Table 2), as it can be affected by space-occupying lesions in the sphenoid sinus (15) and by large nasopharyngeal tumors. This structure was only assessable, particularly in bilateral CI condition or at oblique angles ipsilaterally, after magnet explantation. DWI was only assessable as category 2 for unilateral 135° condition for the contralateral frontal sinus. Except for this, all other DWI images of the paranasal sinuses were not assessable due to the artifacts (category 3 and 4—see Table 2).

### Nasopharynx

**Figure 4:**
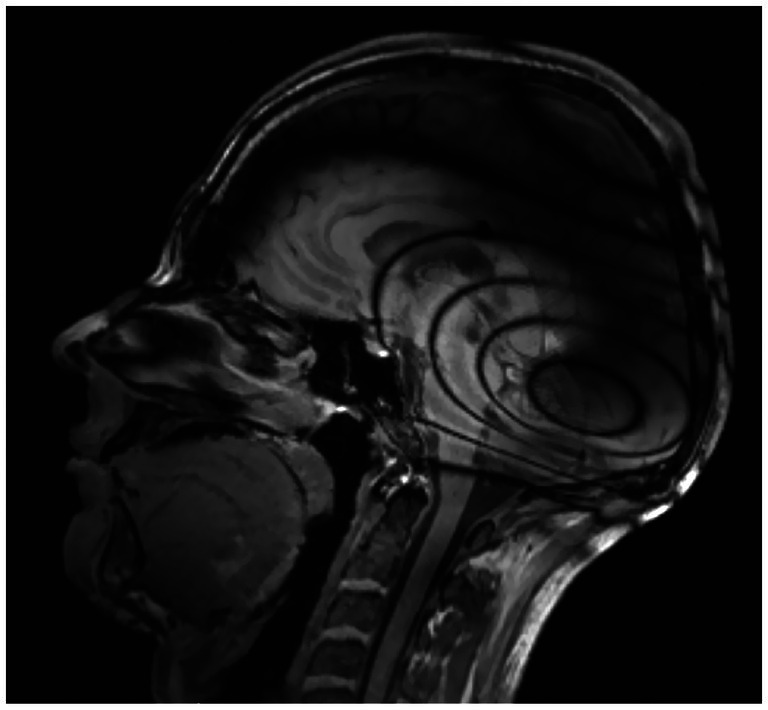
T1-weighted sequence in a bilateral CI with a magnet positioned at a 135° angle demonstrating that the nasopharynx is only partially assessable.

In cases of ipsilateral CI (with and without the magnet *in situ*), the nasopharynx was clearly assessable in the performed sequences [T1-MP-RAGE, T2-TSE, T1-TIRM (Table 2)]. On the contralateral side, the nasopharynx was evaluable in all conditions and sequences. However, with bilateral CIs and the magnet *in situ*, the nasopharynx was not fully assessable due to artifact overlap (see Figure 4). After magnet explantation, visibility was restored. DWI was not assessable at all for the nasopharynx with magnet in situ for ipsi- and contralateral CI condition in all three examined angular positions. The sphenopalatine foramen/pterygopalatine fossa was visible in the ipsilateral CI condition but not in the bilateral CI condition.

## Discussion

### Orbit

In all sequences, except for the DWI, the orbit was visible and assessable (category 1 or 2) under the examined conditions, including in the bilaterally CI condition with the magnet in place. However, the orbital roof was not fully assessable in T1 sequences, especially when the CI was positioned at a 90° angle. The literature emphasizes the importance of T1- and T2-weighted sequences for differentiating the diverse group of orbital lesions (4, 7). Nevertheless, several recent studies have highlighted the utility of DWI in orbital MRI (5–7) for distinguishing between various types of space-occupying lesions. In this case, removing the magnet is essential to make reliable conclusions regarding DWI findings.

### Paranasal sinuses

The results of this study demonstrate the clear and reliable visibility of the maxillary sinus, frontal sinus, ethmoidal cells, and nasal cavity under the evaluated conditions and across the included T1- and T2-sequences. However, the upper ethmoidal cells were partially obscured by artifacts in cases of ipsilateral 90° CI positioning. For a complete evaluation of the ipsilateral portion of the sphenoid sinus in cases of unilateral or bilateral CI, magnet explantation is recommended to ensure a clear and comprehensive assessment.

In both benign and malignant tumors of the paranasal sinuses, MRI is essential not only for planning surgical resection but also for precise radiotherapy planning. Farina et al. (16), along with multiple earlier studies, emphasized that early-stage and recurrent tumors often present with non-specific or absent symptoms, making MRI crucial for identifying dural, skull base, orbital, pterygopalatine fossa, and masticator space infiltration (8, 16–18). Furthermore, DWI has become increasingly important in MRI imaging of inverted papillomas of the paranasal sinuses due to its ability to differentiate purely benign tumors from those that have undergone malignant transformation (19, 20). DWI imaging is not assessable for all included CI conditions with the magnet *in situ* in our study, and therefore explantation of the magnet before MRI is recommended in cases involving DWI.

Due to its specific anatomical location, the clivus was evaluated separately in this study and showed good visibility in T2 sequences. However, in ipsilateral and bilateral CI cases with the magnet in situ, streak-like artifacts interfered with imaging, particularly in T1-weighted sequences. Palsetia et al. (15) and Kunimatsu and Kunimatsu (21) highlighted the importance of T1-weighted imaging for evaluating the clivus, as clival pathologies are best assessed using non-fat-saturated, non-contrast T1-weighted sequences due to the clivus’s central location and fatty marrow content. Tumors of the sphenoid sinus and nasopharyngeal carcinomas can invade the clivus, significantly worsening the disease prognosis (15). Therefore, in such cases, magnet explantation is recommended to ensure sufficient visibility in T1-weighted imaging.

### Nasopharynx

The findings indicate that an ipsilateral CI does not compromise the assessment of either the ipsilateral or contralateral nasopharynx. However, in bilateral CI cases, optimal visibility was only achieved after magnet explantation. As Baba et al. (13) highlighted, MRI is crucial for suspected angiofibroma cases, with preoperative embolization recommended before resection. This tumor, most common in young male adolescents, is the most frequent benign sinonasal tumor, making up 0.5% of all head and neck tumors (13, 22). It may involve the sphenopalatine foramen, pterygopalatine fossa, and skull base (13). Our study demonstrates that magnet explantation is necessary to exclude infiltration of these structures. Nasopharyngeal carcinomas, which are typically treated with a combination of chemotherapy and radiotherapy, require fusion imaging of CT and MRI scans for precise three-dimensional radiation planning (23, 24). In conclusion, to exclude nasopharyngeal masses such as sinonasal tract angiofibromas or nasopharyngeal carcinoma in bilateral CI patients, magnet explantation is strongly recommended.

As outlined in our previous study (3) and supported by the results of this study, artifact sizes are consistent with those reported in earlier studies from other manufacturers (1, 2, 25–27) and can, therefore, be generalized to other CI manufacturers.

### Study limitations

A limitation of this study is the ethical decision not to administer contrast agents, meaning that T1 sequences were examined without gadolinium enhancement. Furthermore, the bandaging of the CI around the head rather than implanting the CI possibly resulting in poorer quality of MRI scans due to artifacts from the skin and subcutaneous tissue. Given that this is a pilot study and no comparable literature exists for the analysis of these specific anatomical regions, alternative MRI sequences may potentially reduce artifact overlap. This should be addressed in future research.

## Conclusion

This study provides comprehensive insights into the predictive value of MRI in assessing and visualizing previously unexamined regions, including the orbit, paranasal sinuses, and nasopharynx in CI patients. The findings emphasize that, particularly for the sphenoid sinus and nasopharynx, prior consultation with the responsible CI center before an MRI examination is strongly recommended. This not only helps optimize and reduce costs but also enhances patient safety and lowers morbidity by avoiding the unnecessary administration of gadolinium-based contrast agents in cases where MRI imaging is not appropriately indicated or performed.

## Data Availability

The raw data supporting the conclusions of this article will be made available by the authors, without undue reservation.
